# Support, technology and mental health: correlates of trainee workplace satisfaction

**DOI:** 10.1007/s40037-019-00555-2

**Published:** 2020-01-17

**Authors:** Vanessa A. Stan, Ricardo Correa, Jessica R. Deslauriers, Semyon Faynboym, Tina Shah, Alik S. Widge, AMA-RFS Task Force on Resident/Fellow Physician Satisfaction

**Affiliations:** 1 Jesse Brown VA Medical Center, Chicago, IL USA; 2 Warren Alpert Medical School, Brown University, Providence, RI USA; 3 Yale University, New Haven, CT USA; 4 Orlando VA Medical Center, Orlando, FL USA; 5 School of Medicine, Indiana University, Indianapolis, IN USA; 6 TNT Health Enterprises, Atlanta, GA USA; 7 University of Minnesota, Minneapolis, MN USA

**Keywords:** Wellness, Burnout, Well-being

## Abstract

**Introduction:**

Low physician workplace satisfaction may negatively impact patient care. Dissatisfaction may begin during residency training, where trainees face lower autonomy and less control over work conditions. The theoretical and empirical literature on trainees is couched mainly in terms of burnout. Theories of satisfaction, a different construct, are derived from studies of independent physicians. Identifying specific correlates of trainee satisfaction may be a clearer path to preparing a sustainable physician workforce.

**Methods:**

We surveyed 3300 residents and fellows (response rate of 7.2% to 46,574 surveys sent) across multiple specialties and institutions in the US. The instrument was adapted from a previous large-scale survey of physician satisfaction, with changes reflecting factors theorized to specifically affect trainee satisfaction. We applied generalized linear regression to identify correlates of higher satisfaction.

**Results:**

A total of 1444 (44%) residents/fellows reported they were very satisfied and 1311 (40%) reported being somewhat satisfied. Factors associated with satisfaction included positive perceptions of supporting clinical staff, the electronic health record, and stability of personal mental health. Surprisingly, a strong negative perception of completing insurance and/or disability forms was also associated with higher satisfaction. Factors often presumed to correlate with satisfaction, such as duty hours, debt load, and specialty, did not show significant associations.

**Discussion:**

Multiple workplace factors are correlated with trainee satisfaction, but they are not the factors (such as financial debt) that we initially hypothesized. The factors we identified, including clinical staff support and personal mental health, may be targets for further study and/or pilot interventions aimed at improving satisfaction.

**Electronic supplementary material:**

The online version of this article (10.1007/s40037-019-00555-2) contains supplementary material, which is available to authorized users.

## What this paper adds

Physician dissatisfaction with the work environment is associated with poor clinical performance and shorter time in practice. Data on the correlates of satisfaction, however, exist only for independent practising physicians. Residents and fellows are younger and more comfortable with health information technology. They are also subject to unique mandates and limitations on their autonomy. This may cause resident/fellow satisfaction to have different correlates than independent physician satisfaction. In a large US-based survey, we showed that this is true. Our findings could aid the design of interventions to improve resident/fellow satisfaction, which could improve physician workforce sustainability.

## Introduction

Physician satisfaction is increasingly recognized as a key component of workforce planning and healthcare delivery. Satisfied physicians may remain in practice longer, increasing access to care and the return on the public’s investment in their training [1, 2]. They deliver greater continuity of care and their patients appear to be more engaged in their own health management [2–5]. Unfortunately, physician satisfaction in the developed world is decreasing [6]. Efforts to combat dissatisfaction have been advanced by a model of dissatisfaction derived from a large qualitative and survey-based study from the RAND Corporation and the American Medical Association (AMA)[2]. This theory focuses on three primary drivers of satisfaction: the overall health system context, features of the specific practice/institution, and individual characteristics of the physician. In that model, factors associated with greater satisfaction are a perception of providing quality care, autonomy, collegiality, and consistent staff support. Poor experiences with the electronic health record, in contrast, are strongly associated with dissatisfaction [2, 7, 8].

A core limitation of this theory is its derivation from studies of independent practising physicians. Significant, career-limiting dissatisfaction may begin earlier, during residency and fellowship. Trainees may be particularly vulnerable to problems with autonomy and support staff availability, as they generally work under supervision, do not have full control over their work hours, and require more staff support. At the same time, current trainees are from a digitally native generation, and may not find the electronic health record to be challenging. It would be valuable to understand whether trainee satisfaction requires a different theoretical framework, because early dissatisfaction could lead to a long career of sub-optimal practice and may exacerbate physician workforce challenges [1].

**Fig. 1:**
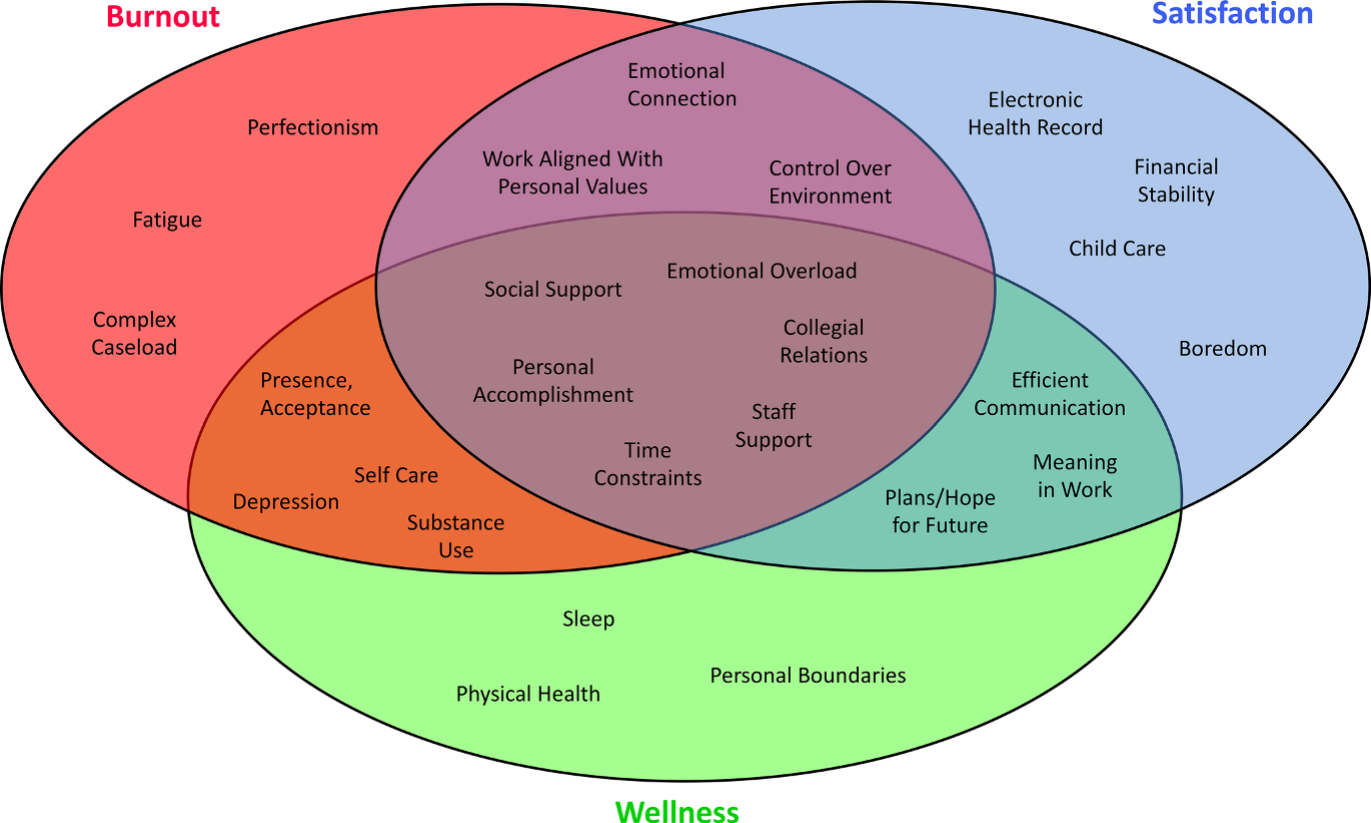
Conceptual illustration of burnout, satisfaction, and wellness. These constructs share many common elements, but each also has unique aspects. For example, wellness includes an emphasis on physical health, whereas satisfaction includes more concepts related to practice logistics. All three constructs influence each other and are substantially correlated

Trainee well-being has been extensively studied, in both single-specialty [9–11] and multi-specialty [12–18] cohorts. The difficulty is that these studies have focused on burnout, not (dis)satisfaction. Burnout is a triad of emotional exhaustion, depersonalization, and a reduced sense of personal accomplishment [19]. Burnout rates are higher among physicians than the general population, and this gap is widening [6]. Burned-out physicians self-report more medical errors [14, 20–25], although the association is inconsistent in prospective studies [26, 27]. Factors associated with burnout include sex/gender [28] and age; [29], being earlier in one’s career [13, 14, 30], financial stress [11, 21, 29, 31], depression [32], uncertainty in the clinical environment [33], poor social/professional support [10, 34–36], and fatigue [32]. Specialty-specific protective factors may exist, e.g. mentoring in neurosurgery [10] or additional income in radiology [11]. In other words, burnout is associated with dissatisfaction [6, 7, 15, 37, 38], but they are not perfect correlates, nor is satisfaction the opposite of burnout (Fig. 1; [39, 40]). Satisfaction is driven more by a sense of belonging and successful completion of a healing mission. To close the gap between these two theoretical views on trainee and physician well-being, we assessed factors linked to both satisfaction and burnout in a national (United States) sample of residents and fellows.

## Methods

### Data collection

We assessed residents/fellows’ work satisfaction using a web-based survey. We began with items included in the RAND/AMA study and the resulting theoretical model [2], including autonomy, support staff relations, and attitudes to electronic health records. Similar to that study, satisfaction itself was assessed as a single item. In some cases, we re-worded items from the prior instrument to make them more relevant to residents/fellows. For example, a question about involvement in financial decisions was re-worded to focus on ‘important departmental decisions’, as residents generally do not have exposure to their departmental finances. We did not use the full instrument from the prior study, but selected questions related to factors that were ultimately found to influence independent physician satisfaction. We then added questions related to factors referenced above as correlated with burnout, e.g. year in training, financial burden, mental health, and self-perception of support from colleagues and other staff. Finally, we added questions regarding factors unique to residents and fellows, such as duty hour limits and formal feedback sessions. These factors are not captured in burnout inventories, and at the time of data collection the Professional Fulfilment Index [25] had not been published. Moreover, at the time of survey development, the majority of the investigators were residents or fellows serving in local, regional, or national leadership roles. The added items were directly derived from the lived experiences of the population under study, which we believed would enhance validity. Finally, we collected demographic data such as age, sex, training environment, and educational debt.

An initial survey instrument was pilot tested for clarity and response consistency among attendees of the American Medical Association (AMA) national meeting (June 2015). We asked attendees to attempt to complete the pilot instrument, and to provide written feedback regarding questions that they found difficult or ambiguous. Based on this feedback, 13 questions were re-worded and one was deleted, with the final wording determined by consensus among the investigators. Responses from the pilot survey were not included in this analysis. The final instrument is included as the online Supplementary Appendix 1, with specific markings of questions that were taken from the RAND/AMA instrument vs. added *de novo*. Data were collected between July and August 2015. A unique survey link was sent to every resident and fellow in the AMA national database. Further, residents and fellows who received the survey were encouraged to invite participation from colleagues in their training institution. Respondents included both allopathic and osteopathic trainees. The study was carried out in accordance with the Declaration of Helsinki: the risk of harm to participants was minimized, anonymity was guaranteed, and informed consent was obtained at the beginning of the survey. All procedures were deemed to be exempt from human subjects review by the Indiana University School of Medicine Institutional Review Board.

We did not pre-specify a sample size. Rather, we aimed to maximize overall data collection within a fixed time period. Our final sample size of 3300 respondents has 80% power to detect effect sizes f^2^ as low as 0.002, even in a multivariate regression using 30 predictors (G*Power version 3.1.8). This is well below the effect sizes actually observed.

### Analysis

Analyses used Stata SE 14 (Stata Corporation, College Station, TX) and R version 3.3.3. We compared sample demographics to the national population as summarized in American College for Graduate Medical Education (ACGME) and Association of American Medical Colleges (AAMC) resources [41, 42], using a binomial exact test with Bonferroni correction for multiple comparisons. We analyzed satisfaction with generalized linear models, using self-reported workplace satisfaction as the dependent variable. We selected a gamma distribution and identity link based on visual review of the distribution. To increase the power to detect specialty-driven differences with a small number of respondents in some specialties, we coded specialty by the ACGME categories of Medical, Hospital-Based, and Surgical Specialties [42]. Questions that asked respondents to rank different issues or priorities were coded dichotomously. That is, for each sub-item within these questions, we created two new variables indicating whether it was in a given respondent’s highest or lowest ranks. ‘Highest’ was defined as placing an item within the top two ranks, while ‘lowest’ was defined as the bottom two ranks.

We built our final regression model by stepwise consideration of all independent variables and minimization of the Akaike information criterion. By definition, this process captures confounds—any confounding variable that explained satisfaction better than its correlates would be automatically selected. We did not attempt a hierarchical analysis, as we were primarily concerned with factors that generalize across trainee populations.

We also conducted a secondary analysis on perceptions of insurance and disability paperwork, based on an unexpected finding (see Results). We performed a multivariate regression using demographic factors as independent variables and perception of paperwork as the dependent variable. We again used a gamma distribution with identity link function.

## Results

### Descriptive statistics

**Fig. 2:**
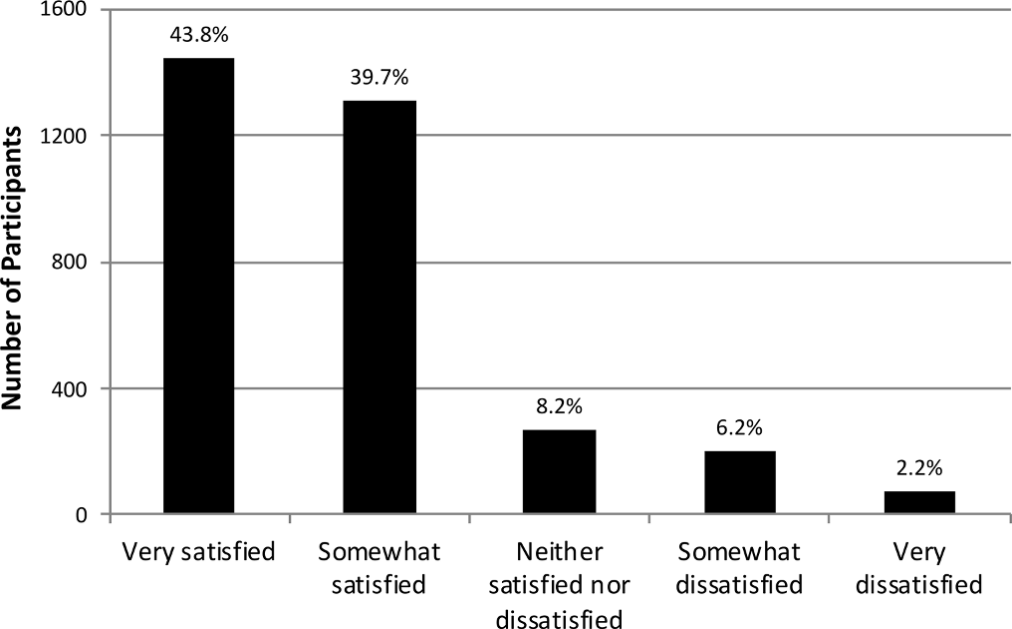
Overall satisfaction with current clinical practice environment, from a sample of 3300 US residents and fellows. The vast majority of resident/fellow respondents were ‘very’ or ‘somewhat’ satisfied

A total of 46,574 surveys were sent, 20,710 were opened, and 3376 were completed (7.2% response rate). Of the responses, 76 were excluded because respondents indicated they were not residents or fellows, leaving 3300 completed surveys for analysis. Compared with national data (Table 1), respondents were more likely to be female, to be carrying educational debt, and to be earlier in training. We under-sampled trainees with low debt and over-sampled at the highest debt levels of $300,000+. There were no respondents from colon and rectal surgery, osteopathic neuromuscular medicine, or nuclear medicine. Of the respondents 84% were very satisfied (40%) or somewhat satisfied (44%, Fig. 2).

**Table 1:** Demographics of the survey sample as compared with national resident/fellow averages

	Survey sample	National population	*P*
*Total responses*	3300	124,409	
*Sex*			
Male	1604 (48.6%)	65,472 (52.6%)	**4.96e‑5**
Female	1650 (50%)	55,021 (44.2%)	**3.83e-10**
Not answered	46 (1.3%)	3916 (3.1%)	**2.40e‑9**
*Level of training*			
PGY1	41.7%	25.8%	**4.23e-86**
PGY2	16.8%	23%	**1.13e-10**
PGY3	17.8%	22%	**3.02e‑8**
PGY4 or above	23.7%	29.2%	**3.41e-11**
*Financial debt*			
None	15.2%	19%	**1.70e‑7**
Under $50,000	13.5%	*	
$50,000–$100,000	10.4%	*	
$100,001–$200,000	20.9%	27.5%	**4.55e-17**
$200,001–$300,000	26.0%	26.7%	1
$300,001–$400,000	10.9%	*	
Over $400,000	3.2%	*	
*Specialty type*			
Medical specialty	1992 (60.4%)	74,759 (60.1%)	1
Surgical specialty	673 (20.4%)	25,419 (19.7%)	1
Hospital-based	631 (19.4%)	24,231 (19.5%)	1

National data are from the 2015 ACGME Data Book and AAMC Debt Fact Card. *P*-values are from a binomial exact test, Bonferroni corrected for multiple testing

**Fig. 3:**
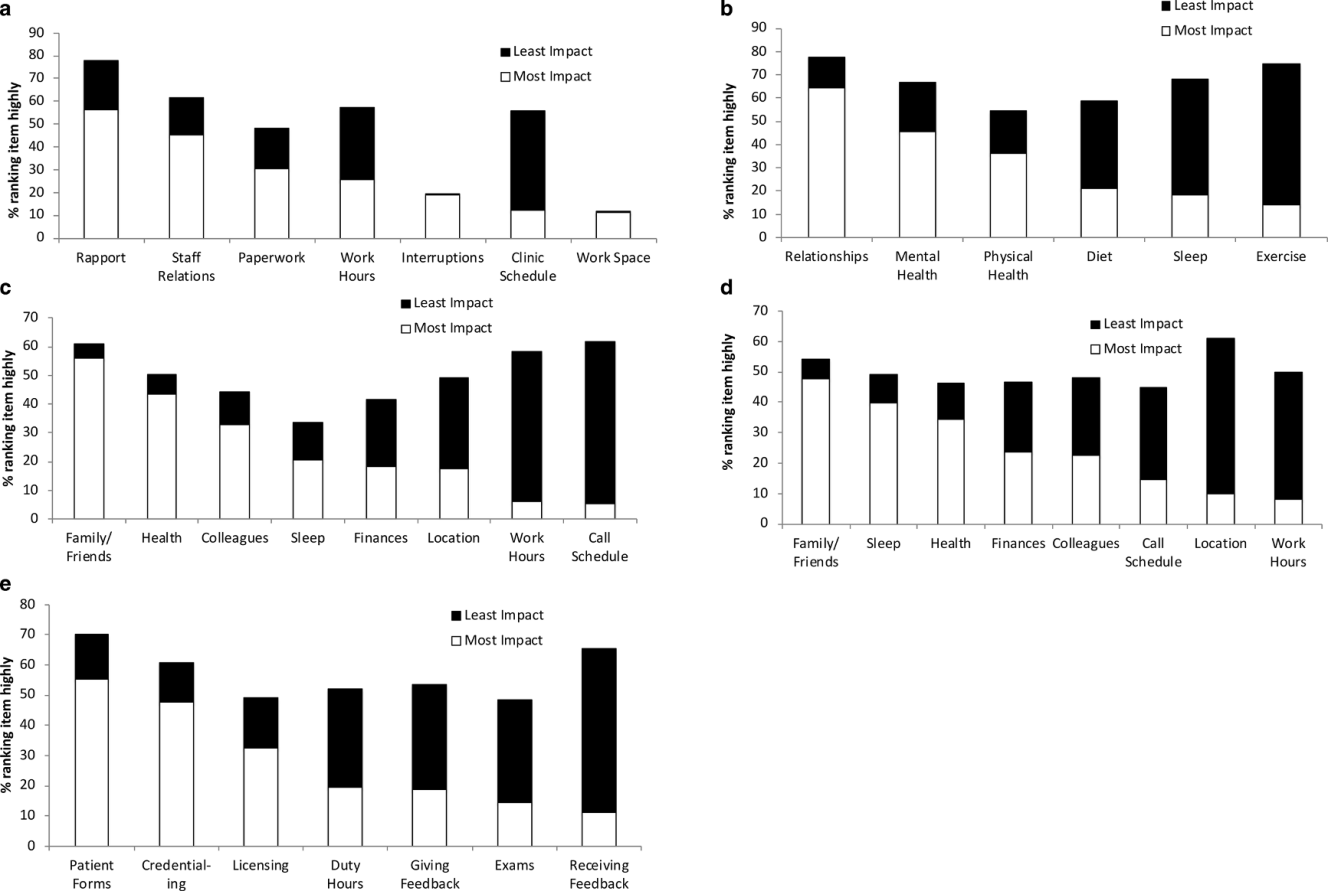
Factors ranked by survey respondents as likely to influence their satisfaction and/or as particularly functional or dysfunctional. Each stacked bar identifies the percentage of respondents who ranked an item as highly impactful (top 2/7) or non-impactful (bottom 2/7). Original item wording is given in questions 7 and 10–13 of the survey (Electronic Supplemental Appendix 1). **a** respondents’ report of workplace factors’ likelihood of impacting satisfaction. **b** factors in respondents’ personal lives that they perceived as (un)stable. **c** factors that respondents felt were high/low life priorities. **d** the degree to which respondents perceived problems in the domains they identified as high priority. **e** the degree to which specific workplace factors interfered with satisfaction and education

Self-reported factors interfering with satisfaction included poor rapport with colleagues and support staff, credentialing/re-credentialing paperwork, and filling out insurance or disability forms for patients (Fig. 3a, e). Sleep and exercise were perceived as particularly unstable (Fig. 3b). Time for friends and family and maintaining physical/mental health were perceived as important for well-being (Fig. 3c). Similarly, family/friend time and sleep were viewed as the most important factors affecting work-life balance (Fig. 2d).

### Model-based predictors of satisfaction

Independent variables that survived stepwise modelling were self-report of: being able to rely on support staff, an electronic health record that improves quality of care, stable mental health, finding feedback from faculty non-interfering, and a high burden of filling out insurance/disability forms for patients (Table 2). Each predicted higher satisfaction when present. Variables that were significant in univariate models (Tables S1–S2 of the online Supplementary Material) but did not survive the stepwise process included: year in training, high debt, perceived autonomy, rapport with physician colleagues and support staff, and variables related to sleep, call, and family/friend time. Factors that were hypothesized to affect satisfaction but that were not significant were: sex (except for non-responders to this item being less satisfied), age, specialty group, poor perceptions of electronic health records, health concerns, sleep concerns, and clinic/service scheduling.

**Table 2:** Correlates of satisfaction (output of generalized linear regression) in a nationally representative sample of residents and fellows

	Coefficient	SE	z	*P* >|z|
Can rely on support staff	0.065	0.003	18.9	5.41e-76
EHR improves quality of healthcare	0.023	0.003	7.2	5.21e-13
Mental health is the most stable	0.173	0.028	−6.2	4.75e-10
Receiving feedback does not interfere with satisfaction	0.096	0.028	−3.4	8.01e-04
Filling out insurance/disability forms completely interferes with satisfaction	0.128	0.028	−4.5	6.10e-06

Regression coefficients have been rescaled to the 0–1 range, and standard errors scaled proportionately. In all cases, a larger coefficient means that an increase in that variable implies greater satisfaction. Self-perception of stable mental health and a perception of high burden from paperwork/disability forms were the strongest predictors of satisfaction

*EHR* electronic health record

### Insurance and disability paperwork as a predictor of satisfaction

The association of insurance/disability paperwork burden with higher satisfaction runs counter to past studies [7, 43, 44]. We examined whether the perceived paperwork load was confounded with other characteristics of the trainee physician. Trainees were more likely to feel that completing insurance and/or disability paperwork interfered with education and work satisfaction if they were female or training in a non-hospital-based specialty (Table 3). More senior trainees and those at a VA/Military site perceived less burden from paperwork. This regression explained 5.84% of the dependent variable’s deviance, suggesting that the majority of paperwork interference is not explained by respondents’ demographics. Perceived interference from insurance/disability forms (item 13) was not related to the overall volume of paperwork (item 7). Endorsing a ‘vast volume of paperwork’ was only weakly correlated with reporting that insurance/disability forms interfere with education and satisfaction (Spearman rho = 0.06, *p* < 0.00044).

**Table 3:** Factors associated with perceived interference in resident/fellow education and job satisfaction from completing disability/insurance paperwork

	Coefficient	SE	*P*(>|t|)
*Sex*			
Female	−0.236	0.067	**4.71e-04**
Not stated	−0.101	0.289	0.726
*Age (base: 25–34 years)*			
18–24 years	−0.097	0.325	0.766
35–44 years	0.227	0.123	0.065
45+ years	0.180	0.367	0.624
*Training setting (base: university)*			
Academic clinic	−0.128	0.131	0.327
Multi-specialty group	0.792	0.413	0.055
Private practice	0.363	0.346	0.293
Community	0.074	0.091	0.413
VA/Military	0.645	0.300	**0.032**
Other	0.556	0.418	0.184
Year in training	0.115	0.023	**5.53e-07**
*Debt (base: no debt)*			
<$50,000	−0.060	0.124	0.630
$50,000–100,000	−0.161	0.130	0.216
$100,000–200,000	0.098	0.115	0.394
$200,000–300,000	−0.004	0.109	0.973
$300,000–400,000	−0.054	0.131	0.679
>$400,000	0.015	0.210	0.944
*Specialty group (base: hospital-based)*			
Medical outpatient	−1.009	0.110	**<2e-16**
Surgical	−0.965	0.124	**8.75e-15**

Negative coefficients correspond to negative emotion, i.e. to finding paperwork more burdensome

## Discussion

We found substantially different correlates of resident/fellow satisfaction compared with independent physicians. Of the factors we studied, only rapport with support staff was a common association between trainees and past studies of independent physicians [2]. Similar to a prior study [15], but in contrast to some frameworks [18, 45], call and sleep items were not significant correlates of satisfaction. Finally, as we initially hypothesized, trainees and independent physicians were discordant in their perceptions of the electronic health record. In past studies, electronic health records drove dissatisfaction when they were difficult to use. Here, electronic health records had a positive effect, significantly increasing satisfaction when they were perceived as improving care. Overall, these results indicate that resident/fellow satisfaction requires its own theoretical model focused on stressors unique to trainees.

Our respondents were earlier in training, more indebted, and more male-female balanced than the national population. They matched, however, the national age and specialty distribution, suggesting that sampling was adequate. Most importantly, none of these demographic factors directly influenced satisfaction in the regression model. This contrasts with studies where age [12–14], gender [46], and specialty [46] were associated with increased trainee burnout. As noted in the Introduction, burnout and workplace satisfaction are not identical constructs, and it may be worth using instruments that measure both in future. For instance, the Professional Fulfilment Index (PFI) was specifically developed to complement the Maslach Burnout Index (MBI) but measures positive-valence satisfaction constructs [25]. Age and career stage may also have non-monotonic effects, with studies finding higher [13, 14] or lower [7] vulnerability depending on the time window studied.

Our conclusions may be limited by a novel survey instrument and by our sample. We did pilot-test the survey prior to data collection, but this remains its first large-scale use. Our instrument overlapped the PFI in items measuring perceived control over the work environment and explicit self-reported satisfaction. Our instrument, however, focused on specific workplace elements, whereas the PFI focuses more on emotional constructs such as self-reported happiness and whether one’s personal values and work assignments are well-aligned. We diverged from both PFI and MBI by omitting items related to exhaustion/depersonalization and instead emphasizing the concrete factors that predicted positive-valence satisfaction. We assessed workplace satisfaction, with slightly different wording than the work-life satisfaction referenced in prior studies [2, 7], although the two should be strongly related. Response rate was relatively low at 7.2%, and over 40% of respondents were new interns. Preferential responding by younger trainees has been common in recent studies [14, 15, 46]. Race and ethnicity data were not collected, limiting our ability to verify reported associations between minority status and trainee satisfaction [12, 46–48].

Given that this was a voluntary survey, respondents might be extremely dissatisfied and seeking an outlet to express dissatisfaction. However, the large numbers of satisfied respondents argue strongly against this. Finally, it was not possible to perform specialty-specific comparisons or subgroup analyses for individual specialties due to the small sample size in several groups. This is a relatively minor limitation, since as noted above, ACGME specialty grouping did not predict satisfaction in the final model. Finally, we note that our sample is specific to the US healthcare system, which has unique economic pressures on physicians and their practice environments. Drivers of trainee satisfaction in more public-health-oriented nations may be different, which would be a very interesting future research question.

Mental health, feedback, and disability/insurance forms all appear more strongly related to workplace satisfaction for trainees compared with independent physicians. Mental health is usually considered part of burnout and wellness constructs, as opposed to being a component of professional satisfaction. Thus, self-perceived stable mental health might either affect or be a consequence of satisfaction. Trainees with unstable mental health might be less able to experience other factors producing satisfaction, or feeling satisfied might lead to greater resilience [33]. The directionality could be studied longitudinally. If mental health drives satisfaction, then trainees with worse self-reported mental health should be less satisfied even very early in residency.

Trainees were more satisfied if they had positive perceptions of feedback. This could mean that improving faculty feedback would improve trainee satisfaction. On the other hand, respondents who are satisfied may be doing well in their clinical programs and receiving mainly positive feedback. Here also, longitudinal data linking frequency and content of feedback to satisfaction measurements might clarify the relationship.

Surprisingly, satisfied respondents were more likely to report that completing disability or insurance forms for patients interfered with education and satisfaction. The demographic variables we collected did not explain the association. Speculatively, over 40% of our sample was in postgraduate year 1, a training stage often associated with ‘scut work’ such as completing patient forms. Younger trainees may be less efficient with such forms. Alternatively, satisfied residents/fellows may perceive fewer problems in other areas, such that paperwork becomes magnified as a complaint. This may relate to our choice of a ranking scheme for some of our survey items. Highly satisfied respondents might not perceive any of our proposed correlates to be a substantial burden, and hence paperwork may simply have been the most burdensome item in a relatively unburdened life.

Importantly, we did not find associations between satisfaction and known resident/fellow stressors such as autonomy, sleep adequacy, debt, and time for friends and family. These negative findings, in a context of adequate statistical power, suggest that satisfaction is not simply a function of lowered stressors.

## Conclusion

Resident and fellow physicians, like independent physicians[2, 49], are generally satisfied with their work environment. Unique factors that may influence trainee satisfaction include mental health, quality of feedback sessions, rapport with support staff, and alignment of electronic health records with care processes. We also found a positive correlation between self-perceived paperwork burden and satisfaction, which may be explained by an unmeasured intermediate variable. Each of these variables is modifiable and measurable at both the departmental and institutional level. We hope that these findings may shape empirical trials of initiatives to improve physician-in-training satisfaction, refocusing the well-being conversation away from burnout.

## Caption Electronic Supplementary Material

Supplementary File 1: Survey instrument

Supplementary File 2: Additional regression outputs
