# Supplementary material for: Support, technology and mental health: correlates of trainee workplace satisfaction
Source: Perspect Med Educ. 2020 Jan 17;9(1):31–40. doi: 10.1007/s40037-019-00555-2 (PMC7012793; doi:10.1007/s40037-019-00555-2)
Supplement: Supplementary file 1 — Survey instrument [file 40037_2019_555_MOESM1_ESM.docx]

**Question derived from the RAND instrument**

**Question derived from the RAND instrument with rewording**

**Question created de novo**

**Resident and Fellow Satisfaction Survey**

*Thank you for sharing your opinions and personal experience with us. To better understand and address challenges that Residents and Fellows face, [BLINDED] invites you to respond to this brief anonymous survey. As always, we will only study and report survey results in the aggregate. Your results will direct [BLINDED] to push for improvements in American resident training and work environment to increase your satisfaction and preparedness for practice.*

**Screener**

1. Are you a…?

- Resident
- Fellow
- Other (Specify)

2. Please indicate which of the following best describes your medical specialty:

- Allergy & Immunology
- Anesthesiology
- Cardiac/thoracic Surgery
- Cardiovascular Diseases
- Dermatology
- Emergency Medicine
- Family Practice/General Practice
- Gastroenterology
- General Surgery
- General Internal Medicine
- Neurology
- Neurological Surgery
- Obstetrics and Gynecology
- Oncology
- Ophthalmology
- Orthopedic Surgery
- Otolaryngology
- Psychiatry
- Pediatrics
- Physical Medicine and Rehabilitation
- Physical and Occupational Therapy
- Plastic Surgery
- Pathology
- Pulmonary Disease
- Radiology
- Radiation Oncology
- Urological Surgery
- Vascular Surgery
- Other (Specify)

**Satisfaction**

3. Overall, how satisfied are you with the environment in which you currently practice?

- Very satisfied
- Somewhat satisfied
- Neither satisfied nor dissatisfied
- Somewhat dissatisfied
- Very dissatisfied

4. What do you think your level of satisfaction will be after completing residency/fellowship?

- Very positive
- Somewhat positive
- Neither positive nor negative
- Somewhat negative
- Very negative

**Practice Environment**

*Now, we’d like to ask you a few questions regarding your practice environment. When answering the following questions, please think about your experiences in the last 3 months.*

5. To what extent do you agree or disagree with the statements, where (1) strongly agree and (5) strongly disagree?

**Scale:**

- Strongly agree (1)
- Somewhat agree (2)
- Neither agree nor disagree (3)
- Somewhat disagree (4)
- Strongly disagree (5)

**Statements:**

1. I am involved in important department decisions
2. I am encouraged to talk openly about what does and does not work in our department
3. My program places an emphasis on my education over hospital duties
4. Hard work is appropriately recognized by my department
5. I can rely on support staff to provide necessary functions and duties to provide high quality patient care
6. There is rapid change in the clinical practice when evidence indicates that we can improve quality/reduce costs
7. My experience working with mid-level providers (NP/PA) has impacted my residency or fellow training experience positively
8. Our leaders provide an environment that is an enjoyable place to work and train
9. I rely heavily on computer-based information when seeing a patient
10. In our institution, our electronic health record improves the quality of care
11. Accessing and entering clinical data by using electronic health records has increased my job satisfaction compared to paper records
12. Using an electronic health record interferes with patient-doctor communication during face-to-face clinical care
13. I understand the principles of medical record documentation related to coding, billing and RVUs

6. Please rank the following workplace characteristics (May not use the same number twice):

*1 Most Important*

*7 Least important*

- Good rapport with my co-physicians and co-residents/fellows
- Easy scheduling of your clinic/service
- Good nursing and support staff
- Reduction in the total work hours
- Decreased volume of "paperwork" (paper or electronic)
- Minimal work interruptions (e.g. non-urgent telephone calls, unscheduled patients)
- Adequate work space (e.g., good office space, good facilities, unlimited supplies)

7. Please rank the following characteristics at your **current** workplace (May not use the same number twice):​

*1 Most likely to impact your overall job satisfaction*

*7 Least likely to impact your overall job satisfaction*

- Poor rapport with the physicians and other residents/fellows that you work with
- Inconsistent scheduling of your clinic/service
- Non-optimal professional relations with support staff
- Increase in the total hours you work
- Vast volume of "paperwork" (paper or electronic)
- work interruptions (e.g., non-urgent telephone calls, unscheduled patients)
- Poor work space (e.g., poor office space, poor facilities, lack of supplies)

8. What level of autonomy are you provided with to make clinical decisions for your patients?

- Too much
- More than I would like
- The right amount
- Less than I would like
- None at all

9. To what extent were you involved in quality improvement initiatives at your residency or fellow program? Select all that apply.

- I was aware of what was going on in my department
- I helped enroll patients or compile data for the initiative
- I was directly involved in implementing the initiative
- I devised and planned the initiative
- I have not been involved in quality improvement initiatives

**Quality of Life:**

*Next, we’d like to ask you a few questions regarding your personal well-being. When answering, please consider your experiences in the last 3 months.*

10. Please rank the following according to stability in your life (May not use the same number twice):

*1 Most stable*

*6 Least stable*

- Physical Health
- Mental health
- Relationships with others
- Sleep
- Diet
- Exercise

11. Please rank the following priorities (May not use the same number twice):

*1 Most important*

*8 Least important*

- Adequate sleep
- Satisfactory physical/mental health
- Adequate family/friend time
- Low call frequency
- Collegial relationships with colleagues
- Desirable location of residency/fellowship
- Work hour limitations
- Financial stability

12. Please rank the following factors (May not use the same number twice):

*1 Most problematic*

*8 Least problematic*

- Poor sleep
- Poor physical/mental health
- Inadequate family/friend time
- High call frequency
- Work hour limitations
- Strained relationships with colleagues
- Financial instability
- Undesirable location of residency/fellowship

13. Please rank the following workplace tasks (May not use the same number twice):

*1 Completely interferes with education and job satisfaction*

*7 Does not at all interfere with education and job satisfaction*

- Following duty hours
- Receiving feedback from faculty
- Credentialing/re-credentialing paper work
- In-service exams
- State or federal licensing applications
- Filling out insurance/disability forms for patients
- Providing feedback to my program, attending physician(s), co-residents, or medical students

**Preparing for Future Practice**

14. Please rank the following according to their importance in terms of selecting your future job (May not use the same number twice):

1 *Most important*

7 *Least important*

- Location
- Schedule (balancing time with family)
- Practice type
- Interaction with colleagues
- Salary
- Direct patient contact
- Ability to be involved in research

15. Thinking about how the future of healthcare delivery will affect your future practice, please rate your level of concern, where (1) is very concerned and (5) is not at all concerned.

- Alternate payment models (less focus on fee for service)
- More stringent Maintenance of Certification (MOC) and licensing requirements
- Adhering to appropriate use criteria in caring for your patients
- Medicaid expansion
- Increasing trend of hospital employment and shrinking of private practice
- Increased reliance on mid-level providers
- Medical liability/defensive medicine pressures

16. In which of the following settings would you most likely want to practice after residency/fellowship? Select all that apply.

- Physician’s office, solo practice
- Physician’s office, single specialty group practice
- Multi-specialty group practice or clinic
- Academic
- Hospital, non-teaching
- Urgent care facility
- Other (Specify)

17. Please indicate to what extent you agree or disagree with the statements, where (1) strongly agree and (5) strongly disagree.

**Scale:**

- Strongly agree (1)
- Somewhat agree (2)
- Neither agree nor disagree (3)
- Somewhat disagree (4)
- Strongly disagree (5)

**Statements:**

- My level of medical school related debt has a major influence on my future practice model choice
- I am prepared to negotiate job contract terms
- I am prepared to manage the business side of my practice

18. How **satisfied** are you with the amount of education your program provided related to the business side of medicine?

- Very satisfied
- Somewhat satisfied
- Neither satisfied nor dissatisfied
- Somewhat dissatisfied
- Very dissatisfied

**Demographics**

D1. Are you…?

- Male
- Female
- Prefer not to answer

D2. What is your age?

- 18-24 years old
- 25-34 years old
- 35-44 years old
- 45+

D4. Which of the following best describes your primary training setting?

- Academic university
- Academic clinic
- Multi-specialty group practice
- Private practice
- Community
- VA/military
- Other

D5. What is your PGY level of training?

- PGY 1
- PGY 2
- PGY 3
- PGY 4
- PGY 5
- PGY 6
- PGY >=7

D6. How much financial debt did you have after graduating from medical school?

- Less than $50,000
- $50,000 to $100,000
- $100,001 to $200,000
- $200,001 to $300,000
- $300,001 to $400,000
- More than $400,000
- None

C1. What, if anything, is impacting your satisfaction as a resident/fellow that is not already captured in this survey? [OPEN TEXT]

*Those are all the questions we have for you, thank you for taking the time to participate in our survey to improve satisfaction while in training.*
