# Supplementary material for: Support, technology and mental health: correlates of trainee workplace satisfaction
Source: Perspect Med Educ. 2020 Jan 17;9(1):31–40. doi: 10.1007/s40037-019-00555-2 (PMC7012793; doi:10.1007/s40037-019-00555-2)
Supplement: Supplementary file 2 — Additional regression outputs [file 40037_2019_555_MOESM2_ESM.docx]

**Table S1:** Univariate gamma GLM regressions on categorical demographic variables. The first row of each regression output reflects the base or intercept term; subsequent rows are additive on that intercept. The only demographics terms to reach significance in a univariate analysis were gender and debt. Respondents who did not specify a binary sex and who had the highest debt levels were less satisfied than peers.

|  | | **Univariate Regression On Sex** | | | |
| --- | --- | --- | --- | --- | --- |
|  | | **Coefficient** | **SE** | **z** | **P>\|z\|** |
| Male | | 4.1978 | 0.0247 | 169.91 | 0.00e+00 |
| Female | | -0.0536 | 0.0344 | -1.56 | 0.119 |
| No Response | | -0.5891 | 0.1262 | -4.67 | **3.17e-06** |
|  | |  |  |  |  |
|  | | **Univariate Regression On Age** | | | |
|  | | **Coefficient** | **SE** | **z** | **P>\|z\|** |
| 18-24 years old | | 4.2667 | 0.181 | 23.536 | 3.71e-113 |
| 25-34 years old | | -0.0811 | 0.182 | -0.445 | 0.656 |
| 35-44 years old | | -0.2811 | 0.188 | -1.495 | 0.135 |
| 45 or older | | -0.3333 | 0.247 | -1.352 | 0.176 |
|  | | **Univariate Regression On Debt** | | | |
|  | | **Coefficient** | **SE** | **z** | **P>\|z\|** |
| None | | 4.2037 | 0.0446 | 94.155 | 0.00e+00 |
| Under $50k | | -0.0497 | 0.0644 | -0.772 | 0.440 |
| $50-100k | | 0.0515 | 0.0703 | 0.733 | 0.464 |
| $100-200k | | -0.0187 | 0.0582 | -0.322 | 0.748 |
| $200-300k | | -0.0229 | 0.0559 | -0.409 | 0.682 |
| $300-400k | | -0.1785 | 0.0668 | -2.675 | **0.008** |
| Above $400k | | -0.3214 | 0.1001 | -3.212 | **0.001** |
|  | | **Univariate Regression On Specialty** | | | |
|  | | **Coefficient** | **SE** | **z** | **P>\|z\|** |
| Medical | | 4.1882 | 0.0220 | 190.09 | 0.00e+00 |
| Surgical | | -0.0780 | 0.0436 | -1.79 | 0.074 |
| Hospital Based | | -0.0514 | 0.0445 | -1.16 | 0.248 |
|  | | | | | |

Table S2: Univariate regressions of continuous and 0/1 coded variables against overall workplace satisfaction, again as gamma GLMs. Positive coefficients represent increased satisfaction when the independent variable is greater. Intercepts for these models are not shown. Many terms, highlighted in bold, were correlated with satisfaction. Most did not pass the AIC-based model selection. Coefficients are on the scale of the original item, usually 5-point Likert scales for Q4-Q8 and binary coding for Q10-Q13.

| **Univariate Regressions on Continuous Variables** | | | | |
| --- | --- | --- | --- | --- |
|  | **Coefficient** | **SE** | **z** | **P>\|z\|** |
| PGY | -0.0268 | 0.0112 | -2.400 | **0.016** |
| Q4 Future Satisfaction | 0.7452 | 0.0141 | 52.722 | **0.00e+00** |
| Q5 Open Culture | 0.4706 | 0.0123 | 38.276 | **1.47e-264** |
| Q5 Education Emphasis | 0.4763 | 0.0125 | 38.008 | **1.71e-261** |
| Q5 Hard Work Recognized | 0.5311 | 0.0123 | 43.219 | **2.87e-322** |
| Q5 Can Rely on Support Staff | 0.3967 | 0.0146 | 27.125 | **4.06e-146** |
| Q5 Enjoyable Environment | 0.6028 | 0.0115 | 52.215 | **0.00e+00** |
| Q5 EHR Improves Care | 0.2457 | 0.0154 | 15.975 | **2.42e-55** |
| Q5 EHR Improves Satisfaction | 0.1679 | 0.0145 | 11.572 | **2.26e-30** |
| Q5 EHR Interferes With Care | 0.0124 | 0.0143 | 0.866 | 0.387 |
| Q7 Poor Rapport | 0.1278 | 0.0342 | 3.734 | **1.92e-04** |
| Q7 Inconsistent Scheduling | -0.0373 | 0.0518 | -0.721 | 0.471 |
| Q7 Support Staff Relations | 0.0850 | 0.0343 | 2.476 | **0.013** |
| Q8 Autonomy | 0.2896 | 0.0353 | 8.199 | **3.47e-16** |
| Q10 Mental Health Stable | 0.2535 | 0.0344 | 7.376 | **2.06e-13** |
| Q10 Relationships Stable | 0.1163 | 0.0354 | 3.287 | **0.001** |
| Q10 Sleep Stable | -0.0620 | 0.0437 | -1.419 | 0.156 |
| Q10 Exercise Stable | -0.0784 | 0.0485 | -1.617 | 0.106 |
| Q11 Sleep Important | -0.1166 | 0.0413 | -2.822 | **0.005** |
| Q11 Health Important | -0.0228 | 0.0344 | -0.663 | 0.507 |
| Q11 Family/Friend Time Important | 0.1391 | 0.0342 | 4.063 | **4.96e-05** |
| Q11 Low Call Important | -0.2200 | 0.0716 | -3.074 | **0.002** |
| Q12 Health Problematic | -0.0539 | 0.0358 | -1.506 | 0.132 |
| Q12 Family/Friend Time Problematic | 0.0682 | 0.0342 | 1.995 | **0.046** |
| Q12 Location Problematic | -0.0998 | 0.0562 | -1.776 | 0.076 |
| Q13 Receiving Feedback Does Not Interfere | 0.2562 | 0.0340 | 7.532 | **6.43e-14** |
| Q13 Disability Paperwork Interferes | 0.1801 | 0.0342 | 5.268 | **1.47e-07** |
